# Dissemination of extensively drug-resistant NDM-producing *Providencia stuartii* in Europe linked to patients transferred from Ukraine, March 2022 to March 2023

**DOI:** 10.2807/1560-7917.ES.2024.29.23.2300616

**Published:** 2024-06-06

**Authors:** Sandra Witteveen, Jörg B Hans, Radosław Izdebski, Henrik Hasman, Ørjan Samuelsen, Laurent Dortet, Yvonne Pfeifer, Niall Delappe, Jesús Oteo-Iglesias, Dorota Żabicka, Martin Cormican, Mirco Sandfort, Felix Reichert, Anna K Pöntinen, Martin A Fischer, Nelianne Verkaik, María Pérez-Vazquez, Niels Pfennigwerth, Anette M Hammerum, Søren Hallstrøm, Marta Biedrzycka, Kati Räisänen, Cornelia CH Wielders, Paweł Urbanowicz, Angela de Haan, Karin Westmo, Fabian Landman, Han GJ van der Heide, Simon Lansu, Romy D Zwittink, Daan W Notermans, Aneta Guzek, Viacheslav Kondratiuk, Aidyn Salmanov, Sebastian Haller, Marius Linkevicius, Sören Gatermann, Anke Kohlenberg, Marek Gniadkowski, Guido Werner, Antoni PA Hendrickx

**Affiliations:** 1 Centre for Infectious Disease Control (CIb), National Institute for Public Health and the Environment (RIVM), Bilthoven, The Netherlands; 2 German National Reference Centre for Multidrug-Resistant Gram-Negative Bacteria, Department of Medical Microbiology, Ruhr-University Bochum, Bochum, Germany; 3 Department for Infectious Disease Epidemiology, Robert Koch Institute, Berlin, Germany; 4 Departement of Molecular Microbiology, National Medicines Institute, Warsaw, Poland; 5 National Reference Laboratory for Antimicrobial Resistance, Department of Bacteria, Parasites and Fungi, Statens Serum Institut, Copenhagen, Denmark; 6 Norwegian National Advisory Unit on Detection of Antimicrobial Resistance, Department of Microbiology and Infection Control, University Hospital of North Norway, Tromsø, Norway; 7 Department of Pharmacy, Faculty of Health Sciences, UiT The Arctic University of Norway, Tromsø, Norway; 8 French National Reference Center for Antimicrobial Resistance, INSERM UMR 1184, Paris-Saclay University, Bicêtre Hospital, Assistance Publique des Hôpitaux de Paris, Paris, France; 9 Division of Nosocomial Pathogens and Antibiotic Resistances, Department of Infectious Diseases, Robert Koch Institute, Wernigerode Branch, Wernigerode, Germany; 10 University of Galway, Galway, Ireland; 11 Reference and Research Laboratory on Antibiotic Resistance of the National Center for Microbiology and CIBERINFEC, Instituto de Salud Carlos III, Madrid, Spain; 12 Departement of Epidemiology and Clinical Microbiology, National Medicines Institute, Warsaw, Poland; 13 Department of Biostatistics, Faculty of Medicine, University of Oslo, Oslo, Norway; 14 Department of Medical Microbiology and Infectious Diseases, Erasmus University Medical Center, Rotterdam, the Netherlands; 15 Department of Health Security, Finnish Institute for Health and Welfare, Helsinki, Finland; 16 Department of Microbiology, Public Health Agency of Sweden, Solna, Sweden; 17 Department of Laboratory Diagnostics, Section of Microbiology, Military Institute of Medicine - National Research Institute, Warsaw, Poland; 18 National Pirogov Memorial University, Vinnytsia, Ukraine; 19 Shupyk National Healthcare University of Ukraine, Kyiv, Ukraine; 20 European Centre for Disease Prevention and Control (ECDC), Stockholm, Sweden

**Keywords:** *Providencia stuartii*, dissemination, New Delhi metallo-β-lactamase, Ukrainian patients, war

## Abstract

**Background:**

The war in Ukraine led to migration of Ukrainian people. Early 2022, several European national surveillance systems detected multidrug-resistant (MDR) bacteria related to Ukrainian patients.

**Aim:**

To investigate the genomic epidemiology of New Delhi metallo-β-lactamase (NDM)-producing *Providencia stuartii* from Ukrainian patients among European countries.

**Methods:**

Whole-genome sequencing of 66 isolates sampled in 2022–2023 in 10 European countries enabled whole-genome multilocus sequence typing (wgMLST), identification of resistance genes, replicons, and plasmid reconstructions. Five *bla*_NDM-1_-carrying-*P. stuartii* isolates underwent antimicrobial susceptibility testing (AST). Transferability to *Escherichia coli* of a *bla*_NDM-1_-carrying plasmid from a patient strain was assessed. Epidemiological characteristics of patients with NDM-producing *P. stuartii* were gathered by questionnaire.

**Results:**

wgMLST of the 66 isolates revealed two genetic clusters unrelated to Ukraine and three linked to Ukrainian patients. Of these three, two comprised *bla*_NDM-1_-carrying-*P. stuartii* and the third *bla*_NDM-5_-carrying-*P. stuartii.* The *bla*_NDM-1_ clusters (PstCluster-001, n = 22 isolates; PstCluster-002, n = 8 isolates) comprised strains from seven and four countries, respectively. The *bla*_NDM-5_ cluster (PstCluster-003) included 13 isolates from six countries. PstCluster-001 and PstCluster-002 isolates carried an MDR plasmid harbouring *bla*_NDM-1,_ *bla*_OXA-10_, *bla*_CMY-16_, *rmtC* and *armA*, which was transferrable *in vitro* and, for some Ukrainian patients, shared by other Enterobacterales. AST revealed PstCluster-001 isolates to be extensively drug-resistant (XDR), but susceptible to cefiderocol and aztreonam–avibactam. Patients with data on age (n = 41) were 19–74 years old; of 49 with information on sex, 38 were male.

**Conclusion:**

XDR *P. stuartii* were introduced into European countries, requiring increased awareness and precautions when treating patients from conflict-affected areas.

**Key public health message**

**What did you want to address in this study and why?**

In bacteria, NDM production can confer drug resistance and NDM-encoding genes can occur on mobile genetic elements, e.g. plasmids. The war in Ukraine has led Ukrainian people to migrate within Europe. Since March 2022, Ukrainian patients with, or affected by, drug-resistant bacteria have been detected in several European countries. We studied 66 NDM-producing strains of *Providencia stuartii* bacteria from Ukrainian patients receiving care in 10 European countries.

**What have we learnt from this study?**

In the European countries considered, NDM-1 and NDM-5-producing *P. stuartii* strains were found predominantly among male patients. The NDM-1 strains were extensively drug-resistant (XDR), and most resistance determinants were localised on a multidrug-resistance (MDR) plasmid. In laboratory experiments, bacteria could exchange the MDR plasmid. For some of the patients, this MDR plasmid was detected in other enteric bacterial species than *P. stuartii*.

**What are the implications of your findings for public health?**

XDR and NDM-producing *P. stuartii* was introduced multiple times in European countries with potential for spread. XDR *P. stuartii* infection leaves limited treatment options. Healthcare professionals should be aware of XDR bacteria linked to migration and evacuation of patients from war regions, and rigorously apply infection prevention and control measures to avoid further transmission.

## Introduction

With the beginning of the war in Ukraine in February 2022, multiple European countries received refugees and medically evacuated patients from Ukraine, including injured civilians and soldiers [1-3]. Since March 2022, the national surveillance programmes of Denmark, Germany, Poland and the Netherlands simultaneously noted an increase of multidrug-resistant (MDR) carbapenemase-producing Enterobacterales (CPE) and *Pseudomonas aeruginosa*. These predominantly consisted of New Delhi-metallo β-lactamase (NDM)- and oxacillinase β-lactamase (OXA-48)-producing *Klebsiella pneumoniae* sequence type (ST)147, ST307 and ST395, as well as *Escherichia coli* ST46 and ST405, and *P. aeruginosa* ST773 and ST1047 [1-3]. However, since the beginning of the war, an increase of patients with uncommon opportunistic CPE, including *Proteus* spp. and *Providencia* spp., also occurred in European healthcare systems [1,3].

The genus *Providencia* comprises *P. alcalifaciens*, *P. heimbachae, P. huaxiensis, P. rettgeri*, *P. rustigianii, P. sneebia, P. stuartii,* and *P. vermicola,* of which *P. stuartii* is one of those causing human infections [4-7]. A 2024 study genomically revisiting the classification of the *Providencia* genus showed evidence that *P. thailandensis* represents the same species as *P. stuartii* [8]. Typically, *P. stuartii* is responsible for urinary tract infections, but it is also associated with pneumonia, bloodstream and wound infections. *P. stuartii* is characterised by intrinsic resistance to aminopenicillins, early generation cephalosporins, colistin and tigecycline as well as its ability to acquire antimicrobial resistance (AMR) genes [4-7], including those associated with resistance to last-resort antibiotics, such as carbapenems. In addition, *P. stuartii* has been shown to cause hospital outbreaks and infections worldwide [5-7,9-12], further highlighting its public health relevance.

In early 2023, the Netherlands reported an increase of carbapenemase-producing *P. stuartii* carrying a *bla*_NDM-1_ gene isolated from Ukrainian patients, on the EpiPulse platform of the European Centre for Disease Prevention and Control. This resulted in a collaborative whole-genome sequencing (WGS) and epidemiological investigation involving 10 European countries. The main objectives of this study were to investigate the cross-border clonal dissemination of NDM-producing *P. stuartii* from Ukrainian patients among 10 European countries. In addition, we explored whether *in vivo* and *in vitro* transfer of the plasmid carrying *bla*_NDM-1_ could occur.

## Methods

### Whole-genome sequencing data collection and analysis

The following countries were contacted by the Netherlands to participate in this study: Germany, Poland, Denmark, France, Norway, Ireland, Italy, Greece, Finland, Spain, Belgium, Hungary, Iceland, Sweden, Wales, England and Portugal. Raw sequence data of *P. stuartii* isolates sampled between March 2022 and March 2023 were obtained in the Netherlands (n = 18 isolates) and also collected from Germany (n = 18), Poland (n = 9), Denmark (n = 6), France (n = 4), Norway (n = 4), Ireland (n = 2), Finland (n = 1), and Spain (n = 1). Previously published sequence data from Italy (n = 3) were also included [13]. Belgium, Hungary, Iceland, Sweden, Italy and Wales reported no *P. stuartii* isolates in that period. England and Portugal reported *P. stuartii* isolates but could not share the data and for Greece, no information was available.

Sequence data from eight European countries were assembled using the in-house National Institute for Public Health and the Environment (RIVM) Juno-pipeline (version 2.0.5; GitHub – RIVM-bioinformatics/juno-assembly: Pipeline to process raw sequencing data up to de-novo assembly and the accompanying statistics) and supplemented with four French and three Italian isolates for which pre-assembled sequences were provided.

Sixteen NDM-1 producing isolates from the Netherlands (12 *P. stuartii,* 2 *E. coli* and 2 *Proteus mirabilis* isolates) and four from Denmark (2 *P. stuartii,* 1 *Citrobacter amalonaticus* and 1 *P. mirabilis*) were sequenced with both Illumina short-read sequencing and Nanopore long-read sequencing (Oxford Nanopore Technologies, Oxford, United Kingdom (UK)) as described previously [14]. Sequence data from recently reported NDM-1 plasmids with IncC replicon (n = 26) were downloaded from the National Center for Biotechnology Information (NCBI) database and included in this study for comparison [13,15,16]. Chromosomes and plasmids were reconstructed using Unicycler (v0.5.0) and internationally retrieved NDM-1-IncC FASTA files of plasmids were annotated with BAKTA (v1.6.1, database 4.0).

Conda databases for ResFinder (v4.1.11) and PlasmidFinder (v2.1.6) from the Center for Genomic Epidemiology were used for the identification of resistance genes and plasmid replicons, respectively. A threshold of 95% was used for identity and 60% for the minimum length for both ResFinder and PlasmidFinder. For the gene *cmlA*, AMRFinder (v3.11.11) was used with the same threshold, since it gave a more detailed description of the gene than ResFinder.

### Whole-genome multilocus sequence typing and core-genome single nucleotide polymorphism analyses

A whole-genome multilocus sequence typing (wgMLST) scheme specific for *P. stuartii* was designed in SeqSphere v8.3.3 (Ridom). The annotated chromosome sequence of *P. stuartii* isolate with GenBank accession number CP014024.2 was used as seed genome. Four other isolates (GenBank accession numbers: AP022374.1, CP027398.1, CP044076.1 and CP071068.1) were used as query genomes. This process yielded a wgMLST scheme comprising 3,079 core genes and 665 accessory genes. To assess the specificity for *P. stuartii*, the wgMLST scheme was tested with a set of *Providencia* spp. isolates other than *P. stuartii* available in the NCBI database. This set included *P. alcalifaciens* (GenBank accession numbers: NZ_CP023536, NZ_CP059346, NZ_CP084296, NZ_LS483467 and NZ_OU659118), *P. heimbachae* (GCA_011754515, GCA_026172745, GCA_900061445, NZ_LS483422 and NZ_CP028384), *P. huaxiensis* (GCA_002843235, GCA_017163435, GCA_018067445 and NZ_CP031123), *P. rettgeri* (NZ_AP022371, NZ_CP027418, NZ_CP029736, NZ_CP039844, and NZ_CP109846), *P. rustigianii* (GCA_000156395, GCA_900455105, GCA_900455235, NZ_LR134189 and NZ_LR134396), *P. sneebia* (NZ_CM001773, only 1 isolate available), *P. stuartii* submitted as *P. thailandensis* (GCA_014652175, GCA_018413475 and GCA_023572545) and *P. vermicola* (GCA_020381325, GCA_029542345, NZ_CP048796, NZ_CP097327 and NZ_CP116222). Finally, we tested 68 *P. stuartii* sequences from the NCBI Reference Sequence (RefSeq) collection. The allelic profiles were then imported into BioNumerics (v8.1.1, Applied Maths, Sint-Martens-Latem, Belgium) to assess genetic relationships between isolates, which were visualised in a minimum spanning tree (MST). Missing alleles were ignored and not counted as allelic differences. In this study, a genetic cluster was defined as ≥ 3 isolates from two or more European countries that differ by ≤ 15 wgMLST alleles (15/3,744 = 0.4% difference); Refseq clusters are excluded. A core-genome single nucleotide polymorphism (cgSNP) analysis was performed in addition to wgMLST analysis. In CLC genomics workbench v23.0.2 (Qiagen), the reads of the isolates were mapped against the NZR-82106 reference strain (SRA accession number: SAMN37519273). Finally, basic variant detection was conducted to create a SNP tree and SNP matrix to compare the wgMLST analysis with cgSNP analysis.

### Antimicrobial susceptibility testing 

Five *bla*_NDM-1_-carrying *P. stuartii* isolates from one cluster (PstCluster-001: RIVM_C047487, RIVM_C048166, RIVM_C048667, RIVM_C048692 and RIVM_C048758), were tested at the Erasmus MC, Rotterdam, the Netherlands by Vitek2 AST-N344 card (bioMérieux, Marcy l'Étoile, France), including amoxicillin–clavulanic acid, ampicillin, cefotaxime, cefoxitin, ceftazidime, cefuroxime, ciprofloxacin, colistin, fosfomycin, gentamicin, imipenem, meropenem, nitrofurantoin, piperacillin–tazobactam, tobramycin, trimethoprim, and trimethoprim–sulfamethoxazole. Furthermore, broth microdilution (BMD) was performed with EUMDROXF (Sensititre, Thermo Fisher Scientific), including amikacin, aztreonam, cefepime, ceftazidime–avibactam, ceftolozane–tazobactam, colistin, eravacycline, fosfomycin + glucose-6-phosphate, imipenem, imipenem–relebactam, meropenem, meropenem–vaborbactam, piperacillin–tazobactam, tigecycline and tobramycin. Susceptibilities to cefiderocol were tested by disk diffusion and BMD (CompASP, Liofilchem). Gradient test strips (all from Liofilchem) were used for testing susceptibility of aztreonam–avibactam, plazomicin and agar dilution was performed for fosfomycin. All AST methods were performed according to the manufacturer’s instructions. For BMD, the reading of the minimum inhibitory concentration (MIC) was performed with Sensititre Vizion Digital MIC Viewing System (Thermo Fisher Scientific, Waltham, United States (US)) by two independent technicians. The obtained results were interpreted using both European Committee on Antimicrobial Susceptibility Testing (EUCAST) v13.1 clinical breakpoints and the Clinical Laboratory Standard Institute (CLSI) guidelines (M100-ED33:2023 Performance Standards for Antimicrobial Susceptibility Testing, 33rd Edition), if available.

### *In vitro* plasmid conjugation

*P. stuartii* isolate 294–22 (from Germany) was used as a donor strain in a broth mating experiment to test the transferability of the carbapenemase gene *bla*_NDM-1_. The sodium azide-resistant strain *E. coli* J53 Azi^R^ was used as recipient. Transconjugants were selected on Luria–Bertani (LB) agar supplemented with ampicillin (50 mg/L), sodium azide (200 mg/L) and a disc with imipenem (10 µg) in the middle of the plate. Single colonies were further cultivated and tested for antibiotic susceptibility and the presence of resistance genes by PCR. Plasmid sizes of the donor strain and the transconjugants were determined by S1 restriction and pulsed-field gel electrophoresis (PFGE), as described previously [17].

### Epidemiological questionnaire

A questionnaire was sent to focal contact points of participating European countries which had provided sequence data of *P. stuartii* to assess the epidemiological characteristics of the patients. The epidemiological metadata collected for *P. stuartii* isolates and patients from European collections included: country, year and month of sample collection, location of the healthcare institution submitting the sample (city and National Territorial Units for Statistics level 2 region), sample type (screening or clinical sample) and site of sampling for clinical samples (e.g. blood, urine), infection (yes/no), clinical relevance (clinical or screening), status of the patient (inpatient or outpatient), suspected type of acquisition (travel-related, community- or healthcare-associated), patient’s age and sex, travel or hospitalisation and country of travel or hospitalisation in the 12 months before sampling, as well as a suspected epidemiological link to another patient. Furthermore, if applicable, Ukraine-specific information was requested, including date and region of the last stay in Ukraine, medical evacuation and mode of transport, type of Ukrainian patient (military/civilian), and combat-related injury (yes/no).

## Results

### Four international genetic clusters of NDM-producing *Providencia stuartii*

**Figure 1:**
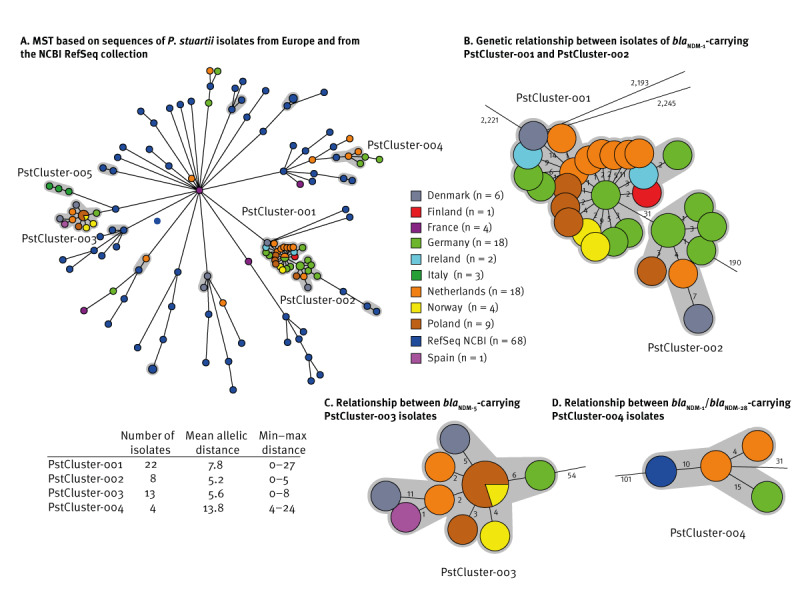
(A) Minimum spanning tree with *Providencia stuartii* RefSeq isolates (n = 68), as well as isolates (n = 66) collected in 10 European countries including from patients linked to Ukraine, and (B–D) focus on individual clusters within the tree, March 2022–March 2023 (n = 134 total isolates)

MST: minimum spanning tree; NCBI: National Center for Biotechnology Information; *P. stuartii*: *Providencia stuartii*; Refseq: NCBI Reference Sequence database; wgMLST: whole-genome multilocus sequence typing.

Whole genome sequences of 66 *P. stuartii* isolates obtained between March 2022 and March 2023 from 10 European countries and complemented with 68 Refseq *P. stuartii* genomes were compared. Four genetic clusters were identified and designated PstCluster-001 to PstCluster-004, respectively (Figure 1A). Another group of three isolates, PstCluster-005, which involved only one country, and did not, as such, fulfil the study definition of a cluster, represented a hospital outbreak in Italy with *P. stuartii* carrying the *bla*_NDM-1_ gene, which was previously described [13]. The largest cluster, PstCluster-001, consisted of 22 *P. stuartii* isolates carrying the *bla*_NDM-1_ gene, which were from seven countries including Denmark, Finland, Germany, Ireland, Norway, Poland, and the Netherlands. A total 19 of the 22 *bla*_NDM-1_-carrying *P. stuartii* isolates were from patients with a known link to Ukraine, while for the three remaining isolates the origin or travels of the patient were unknown. Within this genetic cluster, the mean allelic distance between isolates was 7.5 with a maximum of 27 wgMLST allelic differences between isolates (Figure 1A-D). The eight isolates of PstCluster-002 also carried the *bla*_NDM-1_ gene and showed only 31 wgMLST allelic differences to PstCluster-001, which may indicate a recent divergence from PstCluster-001. PstCluster-002 isolates were found in Denmark, Germany, Poland, and the Netherlands (Figure 1B). For five of the eight isolates, there was a confirmed link to Ukraine, while for the remaining three isolates the origin of the patient was unknown. Isolates from PstCluster-003 carried the *bla*_NDM-5_ gene. This cluster contained 13 *P. stuartii* isolates from six countries (Denmark, Germany, Norway, Poland, Spain, and the Netherlands; Figure 1C). For 12 of the 13 isolates there was a known link to Ukraine, while for one isolate this was unknown. PstCluster-004, consisted of four *P. stuartii* isolates carrying either *bla*_NDM-1_ or the *bla*_NDM-28_ gene, which were from Germany and the Netherlands and also included one RefSeq isolate from Germany (Figure 1D). The NDM-1/NDM-28-producing PstCluster-004 isolates were collected from patients without a known link to Ukraine, but two of these four patients (one in the Netherlands and one in Germany) had previously been hospitalised in Hungary. The three isolates from Italy (PstCluster-005) and two *bla*_NDM-1_-carrying isolates from Denmark, all without a link to Ukraine, and which formed separate groups in the MST, did not relate to the Ukrainian *P. stuartii* isolates or clusters (Figure 1A). Furthermore, 14 *P. stuartii* isolates from France (n = 4), Germany (n = 4), and the Netherlands (n = 6), without an epidemiological link to Ukraine did not cluster, but were dispersed across the MST. Isolates submitted to NCBI as *P. thailandensis* were not divergent from *P. stuartii* and shared the same core genes [8]. Of note, publicly available sequence data for *P. alcalifaciens*, *P. heimbachae*, *P. huaxiensis*, *P. rettgeri*, *P. rustigianii*, *P. sneebia*, and *P. vermicola* isolates failed the in-house *P. stuartii* wgMLST scheme and had less than 11% of the core genes present in the wgMLST scheme. This shows that the wgMLST scheme is specific for *P. stuartii* and that other *Providencia* spp. have a very different core genome. cgSNP analysis on the four genetic clusters was performed and compared with wgMLST analysis, which resulted in an identical distribution of clusters with low cgSNP variations, as illustrated in Supplementary Figure S1.

A. MST based on a *P. stuartii* wgMLST scheme of European *P. stuartii* isolates. Isolates are in different colours depending on the country of detection and the NCBI RefSeq *P. stuartii* sequences are indicated in dark blue. Groups of ≥ 2 isolates that differ by ≤ 15 wgMLST alleles are highlighted with a grey halo. Each circle represents an isolate and a large circle represents > 1 isolate. Number between circles represent the number of allelic differences. In the inset, the number of isolates per cluster is indicated as well as the mean wgMLST allelic distance and the minimum–maximum allelic distance.

B. Genetic relationship between *bla*_NDM-1_-carrying PstCluster-001 and PstCluster-002 cluster isolates from seven and four European countries, respectively.

C. Genetic relationship between *bla*_NDM-5_-carrying PstCluster-003 isolates from six European countries.

D. Genetic relationship between *bla*_NDM-1_/*bla*_NDM-28_-carrying PstCluster-004 isolates from two European countries and a NCBI RefSeq *P. stuartii* isolate.

The 10 European countries where isolates were collected for the study included Denmark, Finland, France, Germany, Ireland, Italy, the Netherlands, Norway, Poland, and Spain.

### Resistomes and antimicrobial susceptibility of *Providencia stuartii* cluster isolates

**Figure 2:**
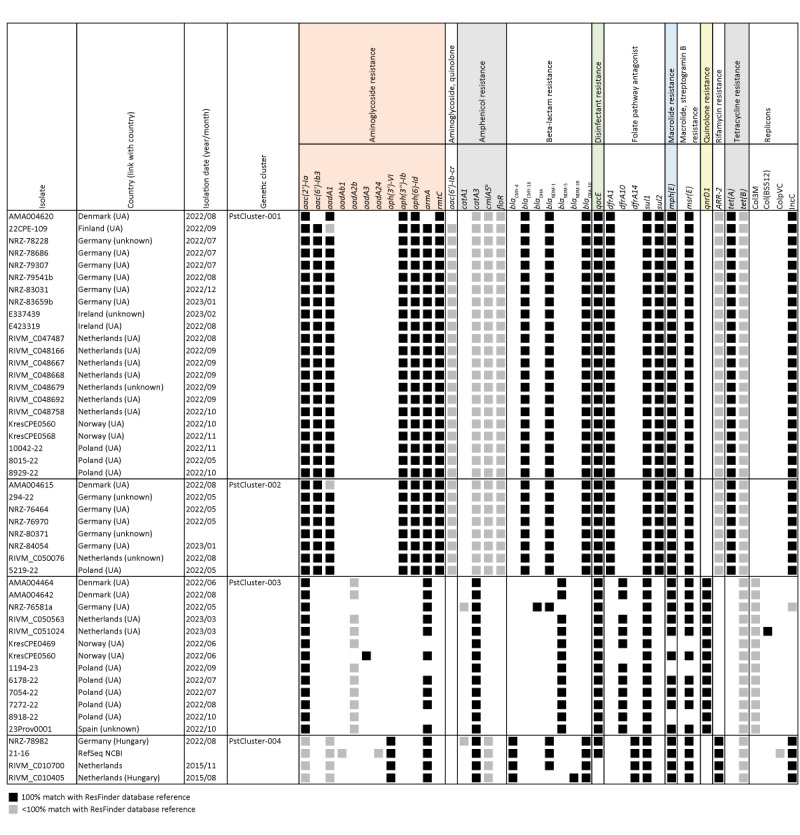
Resistomes of *Providencia stuartii* strains isolated in 10 European countries, which belonged to a cluster according to the study^a^, March 2022–March 2023 (n = 47 total isolates)

AMR: antimicrobial resistance; RefSeq NCBI: Reference Sequence database at the National Center for Biotechnology Information; UA: Ukraine; wgMLST: whole-genome multilocus sequence typing.

^a^ In this study, a genetic cluster was defined as ≥ 3 isolates from two or more European countries that differ by ≤ 15 wgMLST alleles (15/3,744 = 0.4% difference). The 10 European countries where isolates were collected for the study included Denmark, Finland, France, Germany, Ireland, Italy, the Netherlands, Norway, Poland, and Spain.

^b^ This gene is found with AMRFinder instead of ResFinder.

The *P. stuartii* cluster isolates carried AMR genes with predicted resistances for multiple classes of antibiotics and disinfectants (quaternary ammonium compounds) (Figure 2).

Cluster isolates are indicated on the left and the presence of AMR genes or genes predicted to mediate resistance to disinfectants is indicated as black/grey squares. Antibiotic classes and disinfectant (quaternary ammonium compounds) are indicated by different colours above the AMR genes.

Patient isolates with a link to Ukraine contained AMR genes encoding resistance to at least nine types of antimicrobials, including eight different antibiotic classes and disinfectants, while isolates without a link to Ukraine (including the RefSeq isolates) had predicted resistance to an average of six different antibiotic classes; this information can also be viewed in Supplementary Table S1.

PstCluster-001 and PstCluster-002 isolates had nearly identical resistomes with all but one isolate in these clusters carrying 23 AMR genes predicted to encode for multidrug resistance (MDR), including aminoglycosides, quinolones, β-lactams, folate pathway antagonists, tetracyclines and disinfectants (Figure 2) [18]. More specifically, these two clusters were also characterised by the presence of *armA* and *rmtC* genes resulting in high-level pan-resistance to aminoglycosides, the plasmid-mediated AmpC β-lactamase gene *bla*_CMY-16_, the oxacillinase gene *bla*_OXA-10_, the carbapenemase gene *bla*_NDM-1_, and an IncC-type of plasmid replicon. Indeed, AST confirmed that the five tested isolates from PstCluster-001 were extensively drug-resistant (XDR) as shown in Supplementary Table S2 [18]. The isolates were resistant to all tested antibiotics, except for cefiderocol and aztreonam–avibactam, interpreted by either CLSI or EUCAST breakpoints. 

More variable resistomes characterised isolates from PstCluster-003 and PstCluster-004, which harboured nine to 15 AMR genes. Most PstCluster-003 isolates carried *bla*_NDM-5_ (12 of 13 isolates), while PstCluster-004 isolates had *bla*_NDM-1_ (3 of 4 isolates) or *bla*_NDM-28_ (1 of 4 isolates). PstCluster-003 and PstCluster-004 strains carried Col3M or IncC plasmid replicons, respectively, and in contrast to those in other clusters, lacked multiple AMR genes including *rmtC*, and *bla*_CMY-16_. Lastly, the presence of *bla*_CMY-4_ distinguished PstCluster-004 from all other clusters.

### Transfer of the conjugative multidrug-resistant IncC plasmid and comparison with international IncC plasmids

**Figure 3:**
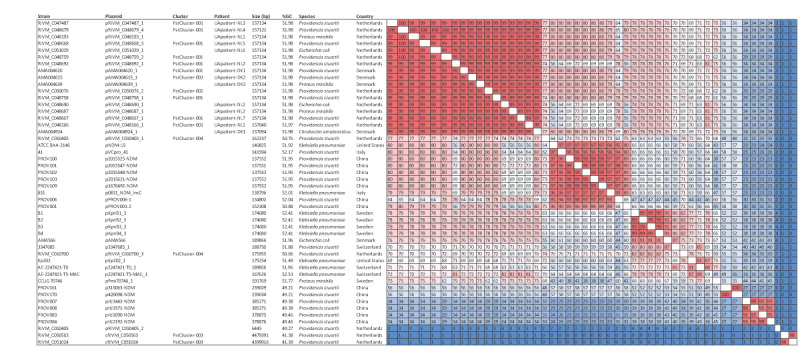
Comparing sequences of *Providencia stuartii* *bla*_NDM-1_-carrying IncC plasmids obtained in the study with those of other reported NDM-1-IncC-plasmids, March 2022–March 2023 (n = 48 isolates)^a^

CPE: carbapenemase-producing Enterobacterales; DK: Denmark; NCBI: National Center for Biotechnology Information; NDM: New Delhi metallo-β-lactamase; NL: the Netherlands; UA: Ukraine.

^a^ Supplementary file 1 contains the NCBI accession and BioProject numbers.

Nanopore long-read sequencing of PstCluster-001 isolates from Denmark (n = 1) and the Netherlands (n = 8), PstCluster-002 isolates from Denmark (n = 1) and the Netherlands (n = 1), and PstCluster-003 (n = 2) and PstCluster-004 (n = 2) isolates from the Netherlands yielded 15 complete IncC plasmid assemblies. The resistance plasmids varied among the four clusters, in line with varying resistomes and plasmid replicons (Figure 2 and Figure 3).

Comparison of *bla*_NDM-1_-carrying IncC plasmids from *Providencia stuartii* cluster isolates from DK and the NL, (including those derived from patients linked to UA) with *bla*_NDM-1_-carrying plasmids from other CPE of Ukrainian patients and internationally reported NDM-1-IncC-plasmids. The G + C content and plasmid size in bp is indicated. The heatmap indicates percentage identity determined by BioNumerics.

**Figure 4:**
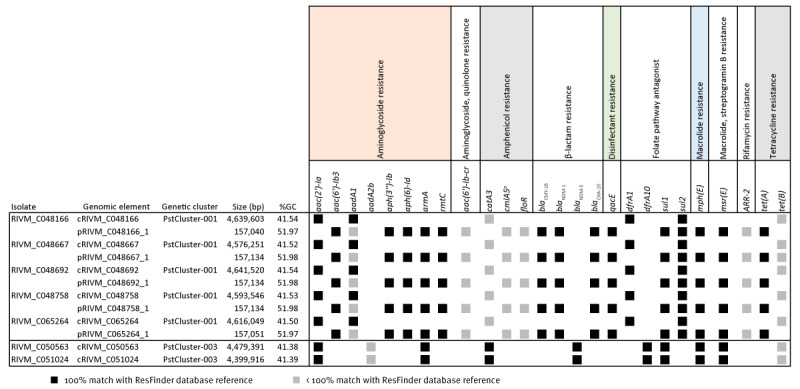
Distribution of antimicrobial resistance genes on plasmids versus chromosomes of *Providencia stuartii* strains, March 2022–March 2023 (n = 7 isolates)

AMR: antimicrobial resistance genes; c: chromosome; p: plasmid.

In PstCluster-001 and PstCluster-002 isolates, most of the *P. stuartii* AMR genes were localised on a novel 157.1-kb plasmid with the IncC-type replicon harbouring AMR genes *bla*_NDM-1_, *bla*_OXA-10_, *bla*_CMY-16,_ *rmtC* and *armA* (Figure 3). The additional AMR genes detected on the plasmid were *aac(6’)-Ib3, aac(6’)-Ib3-cr, aadA1, aph(3”)-Ib, aph(6)-Id, ARR-2, cmlA5, floR, mph*(E), *msr*(E), *qacE, sul1, sul2* and *tet*(A). The *aac(2’)-Ia, catA3, dfrA1,* and *tet*(B) genes were located on the *P. stuartii* chromosome, as were copies of the *aadA1* and *sul2* genes (Figure 4). In PstCluster-003, the NDM-5-encoding gene was localised on the chromosome, along with the other resistance genes (Figure 4).

The presence of AMR genes or genes predicted to mediate resistance to disinfectants is indicated as black/grey squares. Antibiotic classes and disinfectant are indicated by different colours above the AMR genes. The G + C content and plasmid size in bp is indicated.

^a^ This gene is found with AMRFinder instead of ResFinder.

The IncC plasmids from PstCluster-001 and PstCluster-002 isolates displayed a high degree of similarity of 99–100%, had a %G + C content of 51.97–51.98% and were predicted to be conjugative (Figure 3). The best match in the NCBI database using Basic Local Alignment Search Tool (BLAST) was 99.99% identity and 92% query coverage with a *K. pneumoniae* plasmid Kp202 (GenBank accession number: CP041083.1 on 27 Feb 2024) and nine additional BLAST matches with high percentage identity to plasmids from other enteric bacterial species were included in the analyses. Four different Ukrainian patients from Denmark (n = 2, UApatient-DK1 and UApatient-DK2) and the Netherlands (n = 2, UApatient-NL1 and UApatient-NL2) carried, in addition to NDM-1-producing *P. stuartii* isolate, *Proteus mirabilis, C. amalonaticus* and *E. coli* isolates with nearly identical *bla*_NDM-1_-carrying plasmids (Figure 3). One Ukrainian patient (UApatient-NL6) in the Netherlands carried an *E. coli* with the *bla*_NDM-1_-carrying IncC plasmid in the absence of *bla*_NDM-1_-harbouring *P. stuartii*.

**Figure 5:**
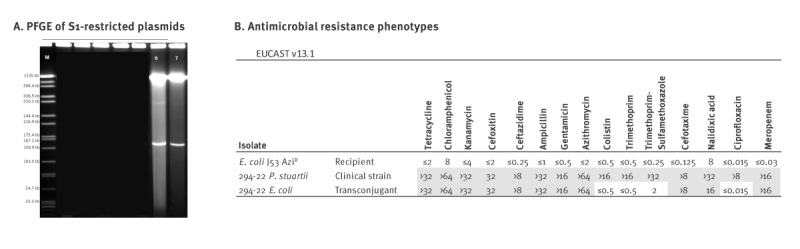
Results of an in vitro trans conjugation experiment at (A) genetic and (B) phenotypic level (n = 3 isolates)

Comparison of the NDM-harbouring plasmids from PstCluster-001 to PstCluster-005 and associated plasmids of CPE from patients in context of recently published NDM-carrying IncC plasmids revealed that these internationally reported plasmids were different [13,15,16] (Figure 3). Furthermore, in an in vitro conjugation experiment, the *bla*_NDM-1_, *bla*_OXA-10_, *bla*_CMY-16,_ *rmtC*, *armA*-carrying MDR plasmid from a PstCluster-002 *P. stuartii* isolate could be transferred to *E. coli* (Figure 5A), thereby transmitting the XDR phenotype to the transconjugant *E. coli* as assessed by AST (Figure 5B).

*E. coli*: *Escherichia coli*; EUCAST: European Committee on antimicrobial susceptibility testing; NDM: New Delhi metallo-β-lactamase; *P. stuartii:* *Providencia stuartii*; PFGE: pulsed-field gel electrophoresis.

A. Evidence (S1-restriction and PFGE) of the transfer of the *bla*_NDM-1_, *bla*_OXA-10_, *bla*_CMY-16_, *rmtC*, *armA*-carrying IncC plasmid from clinical isolate *P. stuartii* 294–22 (lane 6) to recipient *E. coli* J53 Azi^R^ yielding the transconjugant 294–22 *E. coli* (lane 7). Lane M, marker strain *Salmonella* serotype Braenderup H9812 (XbaI-restricted).

B. Transfer of plasmid-mediated antimicrobial resistance phenotypes (grey-shaded) from clinical isolate *P. stuartii* 294–22 to susceptible *E. coli* J53 Azi^R^ recipient strain and yielding the 294–22 *E. coli* transconjugant.

### Epidemiology of patients carrying *Providencia stuartii*

For the Netherlands, most isolates (7/18) belonged to PstCluster-001, while for Germany there was a near equal distribution of *bla*_NDM-1_-carrying isolates among PstCluster-001 (6/18) and PstCluster-002 (5/18). In contrast, Poland and Norway had their isolates roughly split between *bla*_NDM-1_-carrying PstCluster-001 (Poland n = 3 vs Norway n = 2) and *bla*_NDM-5_-carrying PstCluster-003 (Poland n = 5 vs Norway n = 2) isolates.

An epidemiological questionnaire was completed by epidemiologists in nine participating European countries. The focal contact point in Italy did not complete the questionnaire because *P. stuartii* isolates had recently been described [13] and the three corresponding patients had no link with Ukraine.

The characteristics of the 63 patients with *P. stuartii* isolates from the nine countries are shown in the Table. The isolates originated predominantly from male patients. A total of 60% (n = 38/63) were male, 17% (n = 11/63) were female, and for the remaining patients, sex was not specified. Patients with information on age were between 19 and 49 years old for PstCluster-001, between 35 and 66 years old for PstCluster-002 and between 28 and 50 years old for the PstCluster-003. A total of 36 patients with *P. stuartii* isolates belonging to PstCluster-001 (n = 19 patients), PstCluster-002 (n = 5 patients), and PstCluster-003 (n = 12 patients) had a previous documented residence in Ukraine, hospitalisation in and/or a travel link to Ukraine, while for none of the patients of PstCluster-004 and none of the non-cluster *P. stuartii* isolates such link was reported. For six patients from whom *P. stuartii* isolates were obtained in Poland, the sites of the last stay in Ukraine could be retrieved as Lviv (n = 3), Ivano-Frankivsk (n = 1), Kharkiv (n = 1), and Kiev (n = 1). The majority (47/63) of *P. stuartii* isolates were found in clinical samples of patients receiving inpatient care. PstCluster-001 and PstCluster-002 isolates were mostly sampled from patients with wounds (n = 16), while PstCluster-003 and non-cluster isolates were sampled from urine (n = 8).

**Table:** Epidemiological characteristics of patients carrying *Providencia stuartii* in nine European countries, March 2022–March 2023 (n = 63 patients)^a^

Characteristics of patients	PstCluster-				Non-cluster	Total
	001	002	003	004		
Number of patients	22	8	13	3	17	63
Age (range)	19–49	35–66	28–50	21–32	11–74	19–74
Unknown	4	2	10	0	6	22
**Sex**						
Male	13	3	10	2	10	38
Female	4	4	0	1	2	11
Unknown	5	1	3	0	5	14
**Previous residency in, hospitalisation in and/or travel to Ukraine**						
Yes	19	5	12	0	0	36
No	0	0	0	1	6	7
Unknown	3	3	1	2	11	20
**Ukraine region of stay before transition**						
Lviv	2	0	1	0	0	3
Kiev	0	0	1	0	0	1
Kharkiv	0	1	0	0	0	1
Ivano-Frankivsk	1	0	0	0	0	1
Unknown	19	7	11	3	17	57
**Type of patient care**						
Inpatient	17	7	9	2	12	47
Outpatient	0	0	1	0	0	1
Unknown	5	1	3	1	5	15
**Infection**						
Yes	10	6	2	1	4	23
No	7	1	4	1	6	19
Unknown	5	1	7	1	7	21
**Site of infection**						
Wound	7	6	1	1	1	16
Urinary tract	2	0	1	0	2	5
Other	1	0	0	0	1	2
Unknown	12	2	11	2	13	40
**Number of patients per country**						
The Netherlands	7	1	2	2	6	18
Germany	6	5	1	1	5	18
Poland	3	1	5	0	0	9
Denmark	1	1	2	0	2	6
Norway	2	0	2	0	0	4
France	0	0	0	0	4	4
Ireland	2	0	0	0	0	2
Finland	1	0	0	0	0	1
Spain	0	0	1	0	0	1
**Sampling year**						
2013	0	0	0	0	2	2
2014	0	0	0	0	1	1
2015	0	0	0	2	0	2
2017	0	0	0	0	1	1
2018	0	0	0	0	1	1
2019	0	0	0	0	3	3
2021	0	0	0	0	1	1
2022	21	7	11	1	8	48
2023	1	1	2	0	0	4
**Sample type**						
Screening	9	1	7	0	6	23
Clinical	11	7	5	2	5	30
Unknown	2	0	1	1	6	10
**Sampling site**						
Rectum swab	3	0	4	0	3	10
Perineum swab	1	0	0	0	0	1
Wound	9	7	2	2	3	23
Pus	1	0	0	0	0	1
Urine	4	0	4	0	4	12
Blood	0	0	0	0	2	2
Unknown	4	1	3	1	5	14

^a^ The Italian (n = 3) isolates are excluded from this table (n = 63). The nine European countries, from which isolates were included were Denmark, Finland, France, Germany, Ireland, the Netherlands, Norway, Poland, and Spain.

## Discussion

In the participating 10 European countries the occurrence of *P. stuartii* was rare. Through a collaborative WGS and epidemiological data-sharing initiative, we report the cross-border clonal dissemination of NDM-producing XDR *P. stuartii* strains, linked to predominantly male patients from Ukraine to European countries. The total number of isolates likely represents an underestimation of the number of introductions of such XDR strains, since not all European countries who were asked shared WGS and epidemiological *P. stuartii* data for this study. England and Portugal detected *P. stuartii* isolates but could not share the data, some countries did not respond, while other countries such as Belgium, Hungary, Iceland, Sweden and Wales reported that they did not detect any *P. stuartii* isolates. Based on wgMLST, cgSNP and resistome analyses of shared and publicly available WGS data, we concluded that all 30 isolates from PstCluster-001 and PstCluster-002 represent a *P. stuartii* strain carrying *bla*_NDM-1_ and the 13 isolates from PstCluster-003 represent another *P. stuartii* strain, with 12 of these 13 carrying *bla*_NDM-5_. PstCluster-002 has likely evolved from PstCluster-001 since isolates in both these clusters were highly related and carried virtually identical resistomes and IncC MDR plasmids. The origin of these NDM-producing *P. stuartii* strains in Ukraine remains unknown, but three patients shared Lviv as the region in Ukraine before transition. However, information was scarce and there are indications that Lviv served as hub for the transfer of patients to European countries (personal communication by Dr Viacheslav Kondratiuk, 2023). Five hospitals located in different regions in Romania reported an increased number of *P. stuartii* isolates between January 2016 and September 2017, with a high percentage (87%; 67/77) of *P. stuartii* isolates carrying the *bla*_NDM-1_ carbapenem resistance gene [7], suggesting that NDM-1-producing *P. stuartii* may be endemic in some parts of Eastern Europe.

The resistomes of *bla*_NDM-1_-carrying *P. stuartii* belonging to PstCluster-001 and PstCluster-002 predicted the XDR phenotype, and AST performed exemplarily for five PstCluster-001 isolates confirmed this phenotype. Therefore, cefiderocol or the combination of ceftazidime–avibactam plus aztreonam, are proposed as potential last-resort treatment options in case of infection. For fosfomycin, MICs were at the breakpoint or higher, so this antimicrobial agent may only be used when the isolate was tested as susceptible by agar dilution. There was also evidence suggesting that the MDR plasmid carrying the *bla*_NDM-1_, *bla*_OXA-10_, *bla*_CMY-16_, *rmtC*, and *armA* genes could spread *in vivo* from *P. stuartii* to other Enterobacterales species present in the patient and it was demonstrated that transfer could occur *in vitro* from *P. stuartii* to *E. coli*.

To date, secondary transmissions of NDM-producing *P. stuartii* from Ukrainian patients to other patients or to residents in the country of detection has not been reported. This suggests that the infection prevention and control measures in hospitals may have generally been effective. However, a four-patient outbreak with a non-related XDR *P. stuartii* strain occurred in a hospital in Rome, Italy in early 2022, showing *P. stuartii*’s potential to spread [13]. Transmission of *P. stuartii* among hospitalised patients would most likely result in asymptomatic colonisation. This study suggests that both XDR *P. stuartii* and the IncC MDR plasmid are easily disseminated, which warrants close monitoring.

A limitation of this study is the lack of baseline data from the participating European countries, including the occurrence of *P. stuartii* in each country in the past years. Therefore, the relation of the number of *P. stuartii* isolates from patients from Ukraine to the total number of patients from Ukraine, and the frequency of screening for CPE carriage among patients from Ukraine remains unclear. In addition, patient information in the epidemiological questionnaire could not be fully retrieved, and the timing and route of migration or medical evacuations of patients are mostly missing. Another limitation is that conjugation experiments of other resistance plasmids from clusters PstCluster-001, PstCluster-003 and PstCluster-004 were not performed.

## Conclusion

We demonstrated multiple incidents of introduction of XDR *P. stuartii* strains into many European countries, supporting the need for screening patients recently arrived from Ukraine and being admitted to a hospital or another healthcare facility, for MDR and XDR organisms, as well as the early and rigorous application of appropriate infection prevention and control precautions in the institutions offering medical assistance and care to war-injured soldiers and refugees.

## Supplementary Data

733000 Supplementary Figure S1

## Supplementary Data

59000 Supplementary Table S1

## Supplementary Data

15000 Supplementary Table S2

## Supplementary Data

17000 Supplementary file 1
