# Supplementary material for: Dissemination of extensively drug-resistant NDM-producing Providencia stuartii in Europe linked to patients transferred from Ukraine, March 2022 to March 2023
Source: Euro Surveill. 2024 Jun 6;29(23):2300616. doi: 10.2807/1560-7917.ES.2024.29.23.2300616 (PMC11158010; doi:10.2807/1560-7917.ES.2024.29.23.2300616)
Supplement: Supplementary Figure S1 [file 2300616_Supplementary_FigureS1.pdf]

This supplementary material is hosted by Eurosurveillance as supporting information alongside the article "Dissemination of extensively drug-resistant NDM-producing Providencia stuartii in Europe linked to patients transferred from Ukraine, March 2022 to March 2023", on behalf of the authors, who remain responsible for the accuracy and appropriateness of the content. The same standards for ethics, copyright, attributions and permissions as for the article apply. Supplements are not edited by Eurosurveillance and the journal is not responsible for the maintenance of any links or email addresses provided therein

A

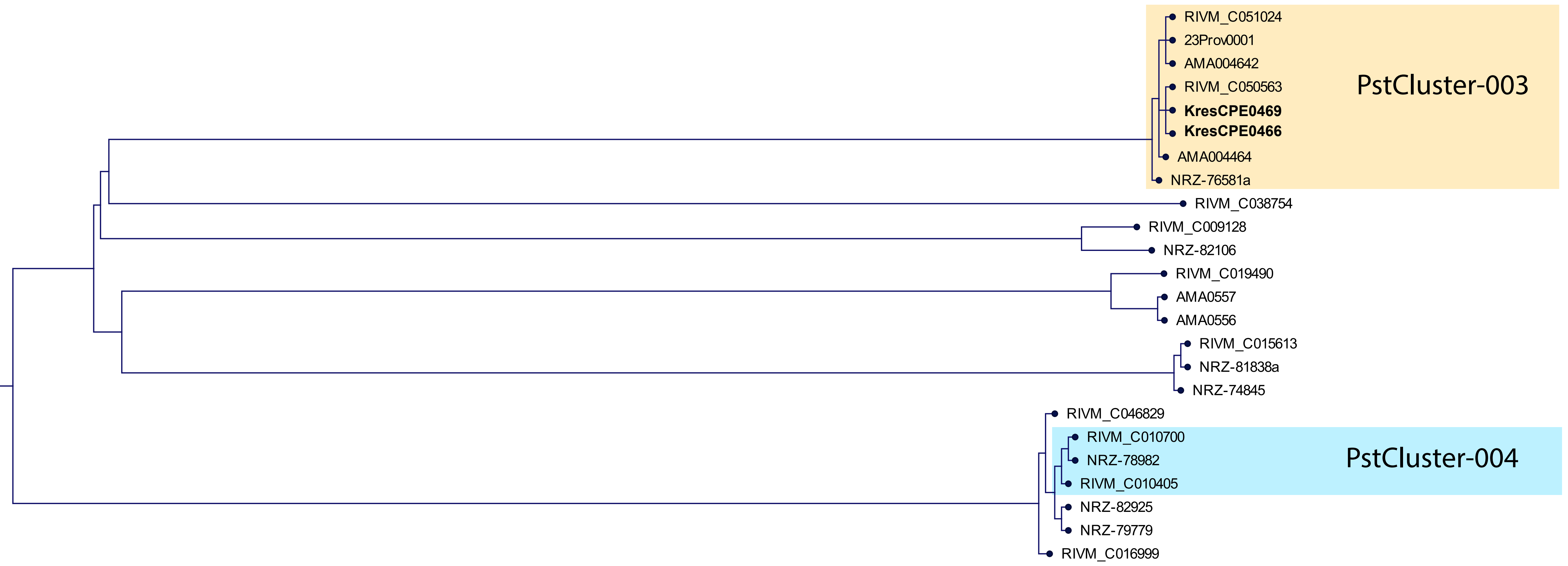

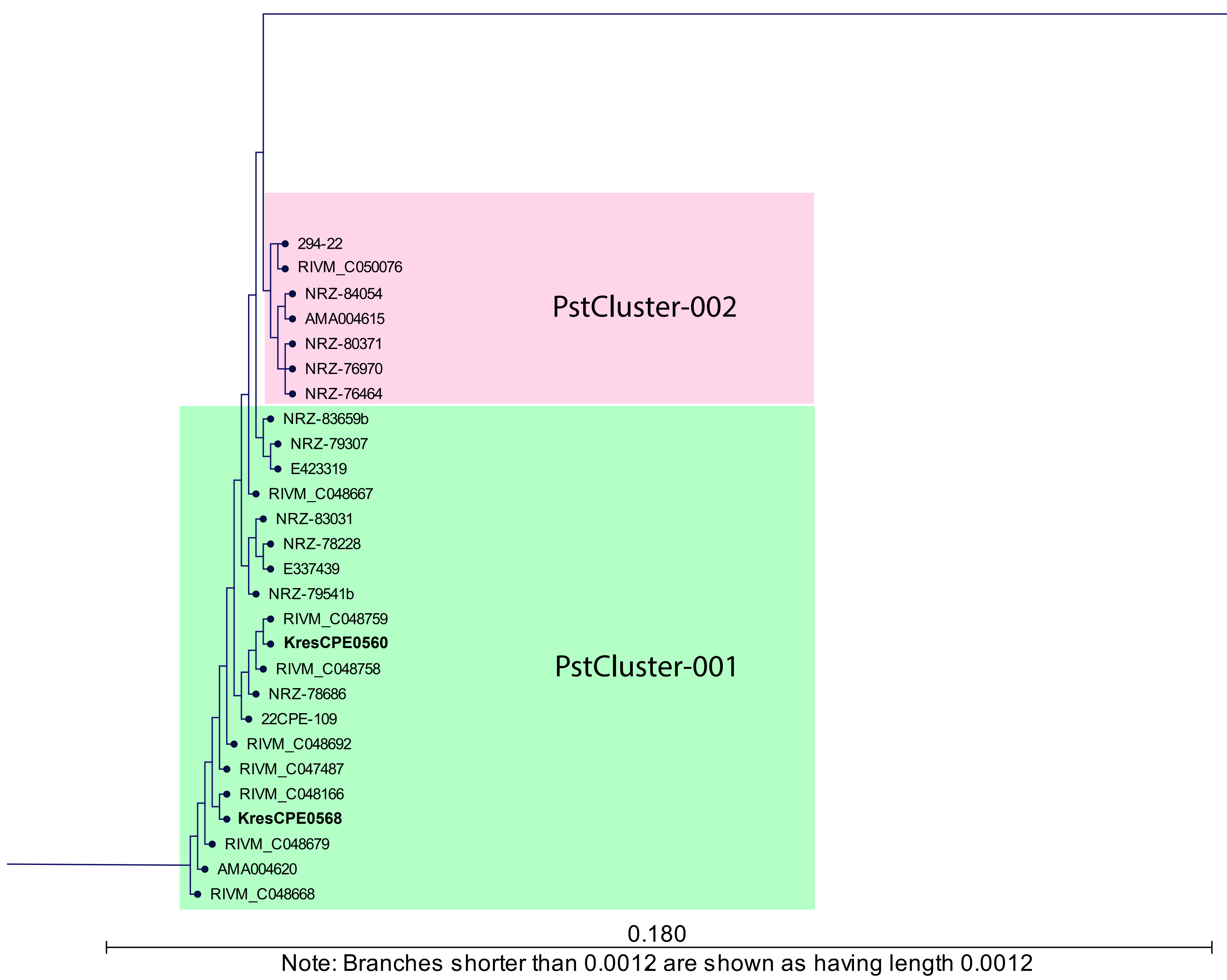

	Min-max distance
PstCluster-001	0 - 18
PstCluster-002	0 - 3
PstCluster-003	0 - 7
PstCluster-004	2 - 12

B

Isolate	PstCluster		1	2	3	4	5	6	7	8	9	10	11	12	13	14	15	16	17	18	19	21	22	23	24	25	26	27	28	29	30	31	32	33	34	35	36	37	38	39	40	41	42	43	44	45	46	47	48	49	50	51
AMA004620	PstCluster-001	1	0	2	4	3	5	3	3	6	7	4	1	2	4	0	2	1	11	8	1	24	23	24	24	24	26	23	#	#	#	#	#	#	#	#	#	#	#	#	#	#	#	#	#	#	#	#	#	#	#	#	#	#
22CPE-109		2	2	0	6	3	7	5	5	8	9	6	3	4	6	2	4	3	11	8	3	26	25	26	26	26	28	25	#	#	#	#	#	#	#	#	#	#	#	#	#	#	#	#	#	#	#	#	#	#	#	#	#	#
NRZ-78228		3	4	6	0	5	7	5	5	8	5	6	5	6	8	4	6	5	15	12	5	28	27	26	26	26	28	27	#	#	#	#	#	#	#	#	#	#	#	#	#	#	#	#	#	#	#	#	#	#	#	#	#	#
NRZ-78686		4	3	3	5	0	6	4	4	7	8	5	4	5	7	3	5	4	10	7	4	27	26	25	25	25	27	26	#	#	#	#	#	#	#	#	#	#	#	#	#	#	#	#	#	#	#	#	#	#	#	#	#	#
NRZ-79307		5	5	7	7	6	0	6	6	5	10	1	6	7	7	5	7	6	16	13	6	27	26	25	25	25	27	26	#	#	#	#	#	#	#	#	#	#	#	#	#	#	#	#	#	#	#	#	#	#	#	#	#	#
NRZ-79541b		6	3	5	5	4	6	0	4	7	8	5	4	5	7	3	5	4	14	11	4	27	26	25	25	25	27	26	#	#	#	#	#	#	#	#	#	#	#	#	#	#	#	#	#	#	#	#	#	#	#	#	#	#
NRZ-83031		7	3	5	5	4	6	4	0	7	8	5	4	5	7	3	5	4	14	11	4	27	26	25	25	25	27	26	#	#	#	#	#	#	#	#	#	#	#	#	#	#	#	#	#	#	#	#	#	#	#	#	#	#
NRZ-83659b		8	6	8	8	7	5	7	7	0	11	4	7	8	8	6	8	7	17	14	7	28	27	26	26	26	28	27	#	#	#	#	#	#	#	#	#	#	#	#	#	#	#	#	#	#	#	#	#	#	#	#	#	#
E337439		9	7	9	5	8	10	8	8	11	0	9	8	9	11	7	9	8	18	15	8	31	30	29	29	29	31	30	#	#	#	#	#	#	#	#	#	#	#	#	#	#	#	#	#	#	#	#	#	#	#	#	#	#
E423319		10	4	6	6	5	1	5	5	4	9	0	5	6	6	4	6	5	15	12	5	26	25	24	24	24	26	25	#	#	#	#	#	#	#	#	#	#	#	#	#	#	#	#	#	#	#	#	#	#	#	#	#	#
RIVM_C047487		11	1	3	5	4	6	4	4	7	8	5	0	3	5	1	3	2	12	9	2	25	24	25	25	25	27	24	#	#	#	#	#	#	#	#	#	#	#	#	#	#	#	#	#	#	#	#	#	#	#	#	#	#
RIVM_C048166		12	2	4	6	5	7	5	5	8	9	6	3	0	6	2	4	3	13	10	1	26	25	26	26	26	28	25	#	#	#	#	#	#	#	#	#	#	#	#	#	#	#	#	#	#	#	#	#	#	#	#	#	#
RIVM_C048667		13	4	6	8	7	7	7	7	8	11	6	5	6	0	4	6	5	15	12	5	26	25	26	26	26	28	25	#	#	#	#	#	#	#	#	#	#	#	#	#	#	#	#	#	#	#	#	#	#	#	#	#	#
RIVM_C048668		14	0	2	4	3	5	3	3	6	7	4	1	2	4	0	2	1	11	8	1	24	23	24	24	24	26	23	#	#	#	#	#	#	#	#	#	#	#	#	#	#	#	#	#	#	#	#	#	#	#	#	#	#
RIVM_C048679		15	2	4	6	5	7	5	5	8	9	6	3	4	6	2	0	3	13	10	3	26	25	26	26	26	28	25	#	#	#	#	#	#	#	#	#	#	#	#	#	#	#	#	#	#	#	#	#	#	#	#	#	#
RIVM_C048692		16	1	3	5	4	6	4	4	7	8	5	2	3	5	1	3	0	12	9	2	25	24	25	25	25	27	24	#	#	#	#	#	#	#	#	#	#	#	#	#	#	#	#	#	#	#	#	#	#	#	#	#	#
RIVM_C048758		17	11	11	15	10	16	14	14	17	18	15	12	13	15	11	13	12	0	15	12	35	34	35	35	35	37	34	#	#	#	#	#	#	#	#	#	#	#	#	#	#	#	#	#	#	#	#	#	#	#	#	#	#
KresCPE0560		18	8	8	12	7	13	11	11	14	15	12	9	10	12	8	10	9	15	0	9	32	31	32	32	32	34	31	#	#	#	#	#	#	#	#	#	#	#	#	#	#	#	#	#	#	#	#	#	#	#	#	#	#
KresCPE0568		19	1	3	5	4	6	4	4	7	8	5	2	1	5	1	3	2	12	9	0	25	24	25	25	25	27	24	#	#	#	#	#	#	#	#	#	#	#	#	#	#	#	#	#	#	#	#	#	#	#	#	#	#
AMA004615	PstCluster-002	21	24	26	28	27	27	27	27	28	31	26	25	26	26	24	26	25	35	32	25	0	1	2	2	2	2	1	#	#	#	#	#	#	#	#	#	#	#	#	#	#	#	#	#	#	#	#	#	#	#	#	#	#
294-22		22	23	25	27	26	26	26	26	27	30	25	24	25	25	23	25	24	34	31	24	1	0	1	1	1	3	0	#	#	#	#	#	#	#	#	#	#	#	#	#	#	#	#	#	#	#	#	#	#	#	#	#	#
NRZ-76464		23	24	26	26	25	25	25	25	26	29	24	25	26	26	24	26	25	35	32	25	2	1	0	0	0	2	1	#	#	#	#	#	#	#	#	#	#	#	#	#	#	#	#	#	#	#	#	#	#	#	#	#	#
NRZ-76970		24	24	26	26	25	25	25	25	26	29	24	25	26	26	24	26	25	35	32	25	2	1	0	0	0	2	1	#	#	#	#	#	#	#	#	#	#	#	#	#	#	#	#	#	#	#	#	#	#	#	#	#	#
NRZ-80371		25	24	26	26	25	25	25	25	26	29	24	25	26	26	24	26	25	35	32	25	2	1	0	0	0	2	1	#	#	#	#	#	#	#	#	#	#	#	#	#	#	#	#	#	#	#	#	#	#	#	#	#	#
NRZ-84054		26	26	28	28	27	27	27	27	28	31	26	27	28	28	26	28	27	37	34	27	2	3	2	2	2	0	3	#	#	#	#	#	#	#	#	#	#	#	#	#	#	#	#	#	#	#	#	#	#	#	#	#	#
RIVM_C050076		27	23	25	27	26	26	26	26	27	30	25	24	25	25	23	25	24	34	31	24	1	0	1	1	1	3	0	#	#	#	#	#	#	#	#	#	#	#	#	#	#	#	#	#	#	#	#	#	#	#	#	#	#
AMA004464	PstCluster-003	28	#	#	#	#	#	#	#	#	#	#	#	#	#	#	#	#	#	#	#	#	#	#	#	#	#	#	0	2	7	3	2	3	1	3	#	#	#	#	#	#	#	#	#	#	#	#	#	#	#	#
AMA004642		29	#	#	#	#	#	#	#	#	#	#	#	#	#	#	#	#	#	#	#	#	#	#	#	#	#	#	2	0	7	3	0	3	1	1	#	#	#	#	#	#	#	#	#	#	#	#	#	#	#	#
NRZ-76581a		30	#	#	#	#	#	#	#	#	#	#	#	#	#	#	#	#	#	#	#	#	#	#	#	#	#	#	7	7	0	8	7	8	6	8	#	#	#	#	#	#	#	#	#	#	#	#	#	#	#	#
RIVM_C050563		31	#	#	#	#	#	#	#	#	#	#	#	#	#	#	#	#	#	#	#	#	#	#	#	#	#	#	3	3	8	0	3	4	2	4	#	#	#	#	#	#	#	#	#	#	#	#	#	#	#	#
RIVM_C051024		32	#	#	#	#	#	#	#	#	#	#	#	#	#	#	#	#	#	#	#	#	#	#	#	#	#	#	2	0	7	3	0	3	1	1	#	#	#	#	#	#	#	#	#	#	#	#	#	#	#	#
KresCPE0469		33	#	#	#	#	#	#	#	#	#	#	#	#	#	#	#	#	#	#	#	#	#	#	#	#	#	#	3	3	8	4	3	0	2	4	#	#	#	#	#	#	#	#	#	#	#	#	#	#	#	#
KresCPE0466		34	#	#	#	#	#	#	#	#	#	#	#	#	#	#	#	#	#	#	#	#	#	#	#	#	#	#	1	1	6	2	1	2	0	2	#	#	#	#	#	#	#	#	#	#	#	#	#	#	#	#
23Prov0001		35	#	#	#	#	#	#	#	#	#	#	#	#	#	#	#	#	#	#	#	#	#	#	#	#	#	#	3	1	8	4	1	4	2	0	#	#	#	#	#	#	#	#	#	#	#	#	#	#	#	#
NRZ-78982	PstCluster-004	36	#	#	#	#	#	#	#	#	#	#	#	#	#	#	#	#	#	#	#	#	#	#	#	#	#	#	#	#	#	#	#	#	#	#	0	12	12	39	38	117	100	#	#	#	#	#	#	#	#	#
RIVM_C010700		37	#	#	#	#	#	#	#	#	#	#	#	#	#	#	#	#	#	#	#	#	#	#	#	#	#	#	#	#	#	#	#	#	#	#	12	0	2	29	28	107	90	#	#	#	#	#	#	#	#	#
RIVM_C010405		38	#	#	#	#	#	#	#	#	#	#	#	#	#	#	#	#	#	#	#	#	#	#	#	#	#	#	#	#	#	#	#	#	#	#	12	2	0	27	26	105	88	#	#	#	#	#	#	#	#	#
NRZ-79779		39	#	#	#	#	#	#	#	#	#	#	#	#	#	#	#	#	#	#	#	#	#	#	#	#	#	#	#	#	#	#	#	#	#	#	39	29	27	0	19	124	107	#	#	#	#	#	#	#	#	#
NRZ-82925		40	#	#	#	#	#	#	#	#	#	#	#	#	#	#	#	#	#	#	#	#	#	#	#	#	#	#	#	#	#	#	#	#	#	#	38	28	26	19	0	123	106	#	#	#	#	#	#	#	#	#
RIVM_C016999		41	#	#	#	#	#	#	#	#	#	#	#	#	#	#	#	#	#	#	#	#	#	#	#	#	#	#	#	#	#	#	#	#	#	#	117	107	105	124	123	0	103	#	#	#	#	#	#	#	#	#
RIVM_C046829		42	#	#	#	#	#	#	#	#	#	#	#	#	#	#	#	#	#	#	#	#	#	#	#	#	#	#	#	#	#	#	#	#	#	#	100	90	88	107	106	103	0	#	#	#	#	#	#	#	#	#
AMA0556		43	#	#	#	#	#	#	#	#	#	#	#	#	#	#	#	#	#	#	#	#	#	#	#	#	#	#	#	#	#	#	#	#	#	#	#	#	#	#	#	#	#	0	1	454	#	#	#	#	#	#
AMA0557		44	#	#	#	#	#	#	#	#	#	#	#	#	#	#	#	#	#	#	#	#	#	#	#	#	#	#	#	#	#	#	#	#	#	#	#	#	#	#	#	#	#	1	0	455	#	#	#	#	#	#
RIVM_C019490		45	#	#	#	#	#	#	#	#	#	#	#	#	#	#	#	#	#	#	#	#	#	#	#	#	#	#	#	#	#	#	#	#	#	#	#	#	#	#	#	#	#	454	455	0	#	#	#	#	#	#
NRZ-74845		46	#	#	#	#	#	#	#	#	#	#	#	#	#	#	#	#	#	#	#	#	#	#	#	#	#	#	#	#	#	#	#	#	#	#	#	#	#	#	#	#	#	#	#	#	0	56	52	#	#	#
NRZ-81838a		47	#	#	#	#	#	#	#	#	#	#	#	#	#	#	#	#	#	#	#	#	#	#	#	#	#	#	#	#	#	#	#	#	#	#	#	#	#	#	#	#	#	#	#	#	56	0	42	#	#	#
RIVM_C015613		48	#	#	#	#	#	#	#	#	#	#	#	#	#	#	#	#	#	#	#	#	#	#	#	#	#	#	#	#	#	#	#	#	#	#	#	#	#	#	#	#	#	#	#	#	52	42	0	#	#	#
NRZ-82106		49	#	#	#	#	#	#	#	#	#	#	#	#	#	#	#	#	#	#	#	#	#	#	#	#	#	#	#	#	#	#	#	#	#	#	#	#	#	#	#	#	#	#	#	#	#	#	#	0	572	#
RIVM_C009128		50	#	#	#	#	#	#	#	#	#	#	#	#	#	#	#	#	#	#	#	#	#	#	#	#	#	#	#	#	#	#	#	#	#	#	#	#	#	#	#	#	#	#	#	#	#	#	#	572	0	#
RIVM_C038754		51	#	#	#	#	#	#	#	#	#	#	#	#	#	#	#	#	#	#	#	#	#	#	#	#	#	#	#	#	#	#	#	#	#	#	#	#	#	#	#	#	#	#	#	#	#	#	#	#	#	0

Number of SNP higher than 1000
